# Prevalence and Determinants of Falls among Older Adults in Ecuador: An Analysis of the SABE I Survey

**DOI:** 10.1155/2013/495468

**Published:** 2013-02-07

**Authors:** Carlos H. Orces

**Affiliations:** Department of Medicine, Laredo Medical Center, 1700 East Saunders, Laredo, TX 78041, USA

## Abstract

The present study based on a nationally representative sample of older adults living in the Andes mountains and coastal region of the country indicates that 34.7% of older adults had fallen in the previous year in Ecuador. Among fallers, 30.6% reported a fall-related injury. The prevalence of falls was higher in women and among older adults residing in the rural Andes mountains. In the multivariate model, women, subjects with cognitive impairment, those reporting urinary incontinence, and those being physically active during the previous year were variables found independently associated with increased risk of falling among older adults in Ecuador. Moreover, a gradual and linear increase in the prevalence of falls was seen as the number of risk factors increased. Falls represent a major public health problem among older adults in Ecuador. The present findings may assist public health authorities to implement programs of awareness and fall prevention among older adults at higher risk of falls.

## 1. Introduction

One-third of people over the age of 65 years who live in the community fall each year; this proportion increases to 50% by the age of 80 years. Although not all falls of older persons are injurious, about 5% of them result in a fracture, and other serious injuries occur in 5% to 10% of falls [1]. Approximately 30% of falls required medical treatment, often resulting in emergency department visits and subsequent hospitalizations, increasing the demand for healthcare services [2]. Previous studies have reported upward trends in fall-related injury hospitalizations and deaths in developed countries [3, 4]. Despite these facts, there is limited information about the epidemiology of falls among older adults in developing countries. 

Reyes-Ortiz et al. (2005) reported that the prevalence of falls among adults aged 60 years or older across seven urban cities in Latin America ranged from 21.6% in Bridgetown, Barbados to 34% in Santiago, Chile [5]. In Brazil, the prevalence of falls found among older adults residing in urban areas was 27.6% [6]. Moreover, among studies in Latin America, the increased risk of falling has been associated with female gender, increased age, high depressive symptoms, functional limitations, diabetes, arthritis, osteoporosis, and urinary incontinence [5–7]. 

In Ecuador, the proportion of persons aged 60 years or older was 6.2% in 1990 and it is expected to reach 11.9% by 2020 and 24.5% by 2050. These demographic changes may markedly increase the number of falls among older adults in the country [8]. Knowledge of the epidemiology of falls may assist public health authorities to implement prevention strategies among individuals at higher risk of falling. Thus, the aims of the present study were to estimate the prevalence of falls and to determine characteristics associated with fall risk among persons aged 60 years or older in Ecuador. 

## 2. Subjects and Methods

The present study was based on cross-sectional data from older adults who participated in the first national survey of Health, Wellbeing, and Aging Study (SABE I), conducted between June and August of 2009. The SABE I survey is a probability sample of households with at least one person aged 60 years or older residing in the Andes mountains and coastal regions of Ecuador. In the primary sampling stage, a total of 317 sectors from the rural areas (<2,000 inhabitants) and 547 sectors from the urban areas of the Coastal and Andes Mountains regions of the country were selected from the 2001 population Census cartography. In the secondary sampling stage, 18 households within each sector were randomly selected based on the assumption that at least one person aged 60 years or older live in 24% and 23% of the households in the Coastal and Andes Mountains, regions, respectively. The objectives of the SABE I survey were to evaluate the health status, cognitive impairment, life style, access and utilization of health care, and functional limitations among older adults in Ecuador. Survey details, including operation manuals, are publicly available [9]. 

### 2.1. Falls Ascertainment

 The prevalence of falls and recurrent falls were assessed by the following questions: “have you fallen in the past year” and “how many times have you fallen in the past year,” respectively. Participants were characterized as recurrent fallers if they had reported two or more falls in the previous year. Participants who answered affirmatively to the question “did you need medical attention as a result of falls” were considered to sustain a fall-related injury. 

### 2.2. Demographic and Health Characteristics

 Age and sex were self-reported. Body height in centimeters and weight in kilograms were measured and the body mass index calculated (Kg/cm^2^). Participants were asked about their living status (alone versus living with others) and area of residence (urban versus rural). The average use of alcohol per week during the previous three months was classified as none, one day, or two or more days per week. 

 Self-reported general health was defined as excellent, very good, good, fair, or poor. Medical conditions were assessed by asking the participants if they had been diagnosed by a physician with hypertension, diabetes mellitus, chronic obstructive pulmonary disease (COPD), arthritis, stroke, or cataracts. Urinary incontinence was defined as having involuntary incontinence of urine during the previous year. 

 Cognitive status was evaluated by the abbreviated Mini-Mental State Examination (AMMSE), which has been validated in the Chilean population. The AMMSE consists of 9 items and has a score from 0 to 19. A score of 12 or less was defined to identify participants with cognitive impairment [10]. The Geriatric Depression Scale was used to evaluate the presence of depressive symptoms. This 15-item scale has been validated in Spanish populations with a sensitivity of 81% and a specificity of 76%. Respondents with a score of 6 or more are considered to have symptoms of depression [11, 12]. The following activities of daily living (ADLs) were included in the present study: walking across a room, dressing, bathing, eating, getting in and out of bed, and using the toilet. Those participants who needed help or were unable to perform one or more of the six ADLs were considered functionally impaired. Physical activity was evaluated by the question “do you regularly exercise such as jogging, dance, or perform rigorous physical activity at least three times weekly for the past year.” Those participants who responded affirmatively were defined as physically active. Lower extremity physical limitation was present if the participants answered affirmatively to any of the following questions: “do you have any difficulty walking a few city blocks” or “do you have any difficulty walking a flight of stairs.” The chair stand test was used to assess lower-limb muscle strength. This test is considered successfully completed if participants are able to stand-up five times from a chair with their arms folded within 60 seconds. Quartiles in seconds were created to analyze the association between lower-limb muscle strength and falls [13]. 

### 2.3. Statistical Analysis

 The chi-square test for categorical variables and the *t*-test for continuous variables were used to compare the characteristics of participants who reported a fall in the previous year and those who did not. Subsequently, those variables statistically significant (*P* value < .05) in the univariate analyses were entered into a logistic regression model to evaluate the independent associations between falls and characteristics of the participants. Results of the regression model are presented as odds ratios (ORs) with their 95% confidence intervals (95% CI). The prevalence of falls was also examined according to the number of independent risk factors found in the multivariate model. Trend in fall prevalence according to the number of risk factors was examined with the chi-square test for trend. To adjust for the multistage sampling design of the SABE I survey, all analyses were weighted by using SPSS, Complex Sample Survey, version 17 software (SPSS Inc., Chicago, IL, USA) to generate national fall prevalence estimates. 

## 3. Results

Of 5,227 participants with complete information on fall status, 37.4% (95% CI, 35.7–39.2) reported to have fallen in the previous year, representing an estimated 445,000 older adults in Ecuador. Recurrent falls (two or more) occurred in 23% (95% CI, 21.5–24.6) of the participants. Moreover, among those who had fallen 30.6% (95% CI, 27.9–33.5) sustained a fall-related injury. 

**Figure 1:**
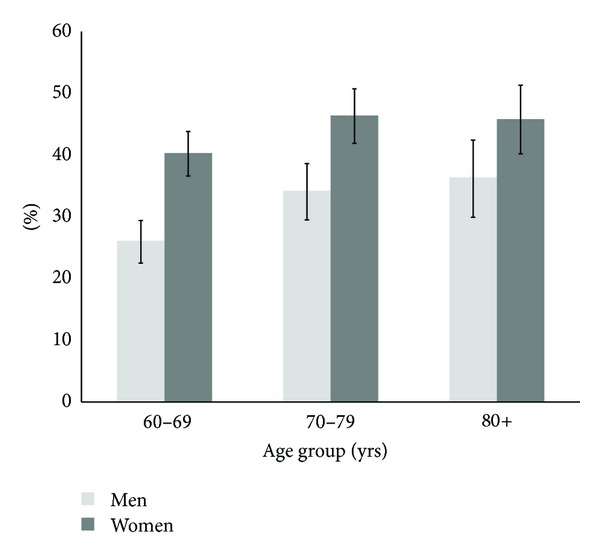
Prevalence of falls among older adults in Ecuador, SABE I.

**Figure 2:**
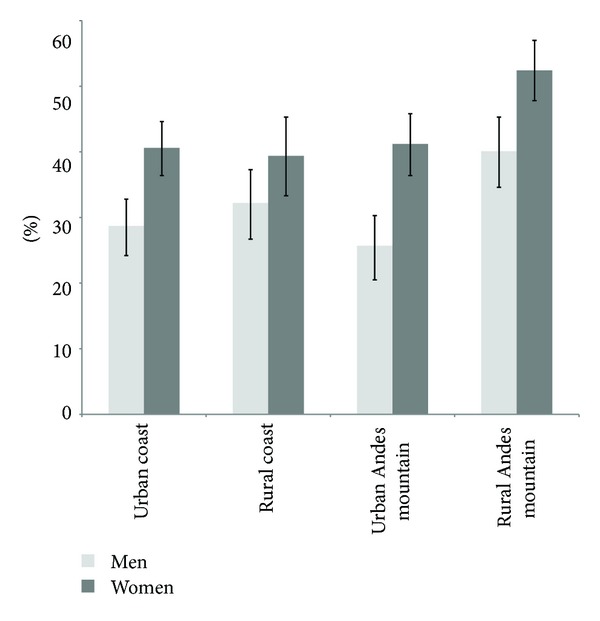
Prevalence of falls among older adults according to area of residence.

The prevalence of falls increased gradually with advancing age and was higher among women (Figure 1). The prevalence of fall-related injuries also increased with age and was higher among women after age of 70 years.  Figure 2 shows the prevalence of falls stratified by gender and area of residence. Overall, the prevalence of falls varies across regions of the country. However, the highest prevalence of falls in both genders was reported among those subjects residing in the rural Andes Mountains. 

 As displayed in Table 1, fallers were more likely to be older, women, residing in rural areas, having poor health status, comorbidities, being less physically active, having functional limitations on the lower extremities and personal ADLs, and having the lowest scores in the chair stand test as compared with non-fallers. In the final multivariate model, women (OR, 1.81; 95% CI, 1.32–2.48), subjects with cognitive impairment (OR, 1.71; 95% CI, 1.18–2.49), those reporting urinary incontinence (OR, 1.58; 95% CI, 1.13–2.22), and being those physically active during the previous year (OR, 1.68; 95% CI, 1.23–2.29) were variables found independently associated with increased risk of falling among older adults in Ecuador (Table 2). Although not statistically significant, a physician's diagnosis of stroke (OR, 0.86; 95% CI, 0.86–2.58) and drinking alcohol ≥2 days per week (OR, 1.47; 95% CI, 0.53–4.04) were also associated with increased risk of falling. 

**Figure 3:**
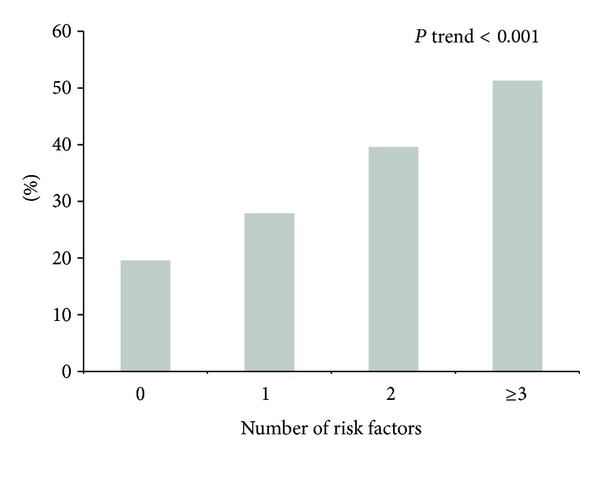
Number of risk factors and prevalence of falls among older adults in Ecuador.

Figure 3 shows the prevalence of falls according to the number of risk factors. Overall, a gradual and linear increase in the prevalence of falls was seen as the number of risk factors increased from 19.6% among persons with no risk factors to 51.3% among those with three or more risk factors (*P* trend < .0001). 

## 4. Discussion

 The present study estimates that 37.4% of community-dwelling older adults fall each year in Ecuador. These results indicate that the prevalence of falls in Ecuador is one the highest reported in the region compared to a previous study of fall prevalence among persons aged 60 years or older in Latin America [5]. Similarly, the prevalence of recurrent falls was higher than those reported in Santiago, Chile, Mexico City, and Sao Paulo, Brazil [5]. 

 The proportion of fall-related injuries among older adults in Ecuador was similar to that reported among persons aged 65 years or older in the USA [14]. Moreover, the increased prevalence of falls and fall-related injuries with advancing age and among women is consistent with other published studies [5, 14–18]. Possible explanations for the higher incidence of fall-related injuries among women have been related to levels of physical activity, muscle weakness and loss of lower body strength, bone mass, circumstances surrounding the fall, and willingness to seek medical attention [17, 18]. 

 Of interest, the highest prevalence of falls occurred among older men and women residing in the rural Andes Mountains. Consistent with this finding, a recent study reported a higher incidence of hip fractures among older adults residing in the Andes Mountains region of the country [19]. Similarly, a study in the USA demonstrated that rural residents have higher fall-related injury rates than urban and suburban residents [20]. Possible explanations for these disparities in fall-related injury may be associated with high-risk occupations such as farming, mining, forestry, and construction among adults residing in rural areas [20]. However, it is unknown whether the same occupational risk factors are present among older adults residing in rural areas of the country. 

 An important finding of the present study was the strong and significant association between cognitive impairment and increased fall risk among older adults in Ecuador. This result is consistent with those from previous research [21–23]. In fact, a systematic review and meta-analysis of twenty-seven studies reported that impairment of global measures of cognition was associated with any fall, serious injuries, and distal radius fractures in community-dwelling older adults. Executive function was also associated with increased risk for any fall and falls with serious injury in institution-dwelling older adults. A diagnosis of dementia of any type was associated with risk for any fall, but not serious injury [23]. Moreover, executive function has been associated to dual tasking and gait variability, supporting the idea that fall risk depends on this function [24]. More recently, results from a prospective cohort study demonstrate that, among community-dwelling older adults, the risk of future falls was predicted by performance on executive function and attention tests conducted five years earlier. In fact, individuals with the lowest score on executive function were more likely to fall sooner and more frequently during the follow-up period [25]. 

 Urinary incontinence was also found an independent and significant risk factor for falls. The present finding is consistent with the results of a recent systematic review [26]. Falls related to incontinence are generally thought to result from loss of balance when rushing to the toilet. However, it is unclear whether incontinence is a primary cause of falls or it is simply a marker of generalized physical frailty [27]. 

 Among the modifiable risk factors, regular alcohol use was associated with increased risk of falling among older adults. Although not statistically significant, there was a 1.4-fold higher risk of falling among older adults who drink on average two or more days per week compared to those who did not. These findings contrast with results from previous cohort studies that reported no significant association between alcohol use and fall risk among older adults [21, 22, 28, 29]. However, low alcohol concentrations among older adults, having considered to be safe for driving, may affect the ability to successfully avoid sudden obstacles in the travel path. Moreover, it is suggested that many of alcohol-related falls are the results of the disruptive effects of alcohol on the online corrections of the ongoing gait pattern when walking under challenging conditions [30]. 

 Several studies have shown that fall risk is closely related to ADLs capability and that difficulty in at least one activity of daily living double the risk of falling [5, 21, 22, 31, 32]. In Ecuador, the risk of falling was 1.2-fold higher among older adults with any impairment in ADLs. This finding confirms the results of a previous study showing that any ADLs limitation among older adults in Latin America and among Mexican-Americans increases significantly the risk of falling [5]. Limitations in ADLs often reflect poor mobility and lower-limb muscle strength, which are major risk factors for falling in older people [15, 33, 34]. In the present study, a significant association between lower-limb muscle strength and falls was found in the unadjusted regression model. However, after adjusting for covariates, a considerable attenuation of the association was observed suggesting that the increased risk of falls among participants with lower-limb muscle weakness was modified by demographic and health characteristics of the subjects. Of relevance, the risk of falling was significantly higher among participants who reported intense regular exercise during the previous year compared to those who did not. This finding may be partly explained by reported changes in postural control among older adults following moderate physical exercise, which may be related to fatigue levels [30]. However, environmental factors and terrain conditions should also be considered as determinants of moderate exercise-related falls among older adults in Ecuador. 

 As previously described by other researchers, a linear increase in the percentage falls is seen as the number of independent risk factors also increases [21, 22]. In Ecuador, the prevalence of falls among older adults with three or more risk factors was 51.3% compared to 19.6% among those with no risk factors. The principal clinical implication of this finding is that the risk of falling may be reduced significantly by modifying even a few risk factors [21]. 

 The present study is the first to provide national estimates on the prevalence of falls among adults aged 60 years and older residing in the coastal and Andes mountains regions of Ecuador and identified demographic and health characteristics associated with increased risk of falling. However, several limitations must be mentioned in interpreting these results. First, the SABE I survey used a 12-month recall period, which is susceptible to recall bias. Second, these findings may also reflect nonresponse bias. Subjects who did not participate in the survey may have been older, frailer, and more likely to have fallen, which would result in underestimating falls. Third, older adults residing in the Amazon region of the country were not included in the SABE I survey. However, they represent only 3.3% of the population aged 60 years and older in Ecuador [35]. Fourth, other known factors associated with increased risk of falling such as gait disorder, orthostatic hypotension, dizziness, use of psychotropic drugs, or use of walking aid were not included in the analysis or collected in the survey. 

 In conclusion, falls represent a major public health problem among older adults in Ecuador. The present findings may assist public health authorities to implement programs of awareness and fall prevention among older adults at higher risk for falls. 

## Figures and Tables

**Table 1:** Demographic and clinical characteristics of survey

	Fallers	Nonfallers	*P* value
Sex (*n*, %)			<.0001
Women	1,177 (62.3)	1,584 (49.2)	
Men	745 (37.7)	1,721 (50.8)	
Age, yrs	72.3 SD 8.8	70.8 SD 8.3	<.0001
BMI (Kg/m^2^)	26.0 SD 4.8	26.0 SD 4.6	.837
Residing in rural areas (*n*, %)	942 (37.9)	1,408 (30.9)	<.0001
Alcohol use (*n*, %)			
None	1,572 (82.2)	2,591 (76.8)	<.0001
1 day	304 (15.0)	634 (21.0)	
≥2 days	45 (2.8)	78 (2.2)	
Self-reported health (*n*, %)			<.0001
Excellent	13 (0.8)	43 (1.6)	
Very good	22 (1.6)	105 (4.0)	
Good	282 (15.6)	730 (24.7)	
Fair	1,033 (53.5)	1,825 (52.9)	
Poor	569 (28.5)	597 (16.8)	
Comorbidities (*n*, %)			
Cognitive impairment	511 (29.0)	568 (17.0)	<.0001
Depression	533 (39.2)	618 (24.4)	<.0001
Hypertension	905 (49.8)	1,382 (43.9)	<.0001
Diabetes	266 (13.9)	396 (12.8)	<.0001
COPD^a^	192 (9.7)	221 (6.9)	<.0001
Arthritis	717 (40.0)	932 (28.0)	<.0001
Stroke	157 (8.9)	165 (4.8)	<.0001
Urinary incontinence	557 (31.8)	614 (19.6)	<.0001
Cataracts	548 (30.4)	803 (24.5)	<.0001
Physical activity (*n*, %)	577 (29.7)	1,078 (32.5)	<.0001
Lower extremity disability (*n*, %)	960 (70.1)	1,255 (62.3)	<.0001
ADLs limitation (*n*, %)^b^	685 (35.0)	713 (21.2)	<.0001
Chair stand test			<.0001
Q1 (4 to 9 sec )	346 (25.0)	842 (30.8)	
Q2 (10 to 11 sec)	313 (22.4)	727 (25.6)	
Q3 (12 to 14 sec)	367 (25.4)	704 (25.5)	
Q4 (unable and ≥15 sec)	385 (27.2)	531 (18.0)	

^a^Chronic obstructive pulmonary disease.

^b^Personal activities of daily living.

**Table 2:** Associations between characteristics of the participants and self-reported falls.

	Unadjusted OR (95% CI)	Adjusted OR (95% CI)
Sex		
Men	1.00 (Reference)	1.00 (Reference)
Women	1.70 (1.46–1.99)	**1.81 (1.32–2.48)**
Age groups, years		
60–69	1.00 (Reference)	1.00 (Reference)
70–79	1.34 (1.13–1.60)	1.13 (0.83–1.55)
≥80	1.40 (1.14–1.72)	0.95 (0.60–1.51)
Live in rural areas		
No	1.00 (Reference)	1.00 (Reference)
Yes	1.36 (1.17–1.58)	1.01 (0.76–1.34)
Alcohol use		
None	1.00 (Reference)	1.00 (Reference)
1 day per week	0.66 (0.54–0.81)	1.16 (0.77–1.73)
≥2 days per week	1.15 (0.70–1.91)	1.47 (0.53–4.04)
Self-reported health		
Excellent	1.00 (Reference)	1.00 (Reference)
Very good	0.81 (0.30–2.18)	0.22 (0.19–2.55)
Good	1.26 (0.57–2.78)	0.90 (0.13–6.20)
Fair	2.03 (0.93–4.40)	1.10 (0.16–7.38)
Poor	3.39 (1.55–7.41)	1.51 (0.22–10.4)
Comorbidities		
Cognitive impairment		
No	1.00 (Reference)	1.00 (Reference)
Yes	1.99 (1.65–2.40)	**1.71 (1.18–2.49)**
Depression		
No	1.00 (Reference)	1.00 (Reference)
Yes	1.99 (1.64–2.42)	1.14 (0.82–1.59)
Hypertension		
No	1.00 (Reference)	1.00 (Reference)
Yes	1.27 (1.09–1.48)	1.19 (0.90–1.58)
Diabetes		
No	1.00 (Reference)	1.00 (Reference)
Yes	1.10 (0.88–1.36)	1.07 (0.71–1.59)
COPD		
No	1.00 (Reference)	1.00 (Reference)
Yes	1.45 (1.10–1.89)	0.98 (0.60–1.59)
Arthritis		
No	1.00 (Reference)	1.00 (Reference)
Yes	1.72 (1.46–2.01)	1.03 (0.76–1.39)
Stroke		
No	1.00 (Reference)	1.00 (Reference)
Yes	1.91 (1.42–2.56)	1.49 (0.86–2.58)
Urinary incontinence		
No	1.00 (Reference)	1.00 (Reference)
Yes	1.91 (1.60–2.28)	**1.58 (1.13–2.22)**
Cataracts		
No	1.00 (Reference)	1.00 (Reference)
Yes	1.34 (1.13–1.59)	0.92 (0.66–1.29)
Physical activity		
No	1.00 (Reference)	1.00 (Reference)
Yes	1.14 (0.96–1.34)	**1.68 (1.23–2.29)**
Lower extremity disability		
No	1.00 (Reference)	1.00 (Reference)
Yes	1.41 (1.16–1.71)	1.20 (0.89–1.61)
Limitations in ADLs		
No	1.00 (Reference)	1.00 (Reference)
Yes	2.03 (1.73–2.39)	1.20 (0.86–1.67)
Chair stand test		
Q1 (4 to 9 sec)	1.00 (Reference)	1.00 (Reference)
Q2 (10 to 11 sec)	1.07 (0.84–1.38)	0.94 (0.63–1.39)
Q3 (12 to 14 sec)	1.23 (0.96–1.57)	0.87 (0.58–1.29)
Q4 (unable and ≥15 sec)	1.86 (1.45–2.38)	1.17 (0.78–1.77)

Bold numbers represent statistical significance in the final multivariate model.
